# Pre-training heart rate variability as a predictor of Air Force Academy completion

**DOI:** 10.1371/journal.pone.0327406

**Published:** 2025-08-07

**Authors:** Yosef Kula, Roy Horosov, Yori Gidron, Aya Ekshtein, Barak Gordon, Zev Iversen, Omer Tehori, Oded Ben-Ari

**Affiliations:** 1 Department of Nursing, Faculty of Social Welfare and Health Sciences University of Haifa, Haifa, Israel; 2 Department of Military Medicine, Faculty of Medicine, The Hebrew University of Jerusalem, Jerusalem, Israel; 3 The Israeli Air Force Aeromedical Center, Medical Corps, Israeli Defense Forces, Ramat-Gan, Israel; 4 Medical Corps, Israeli Defense Forces, Ramat-Gan, Israel; 5 Behavioral Science Center, Israeli Defense Forces, Ramat Gan, Israel; 6 The Adelson School of Medicine, Ariel University, Ariel, Israel; Portugal Football School, Portuguese Football Federation, PORTUGAL

## Abstract

**Introduction:**

Operational pilots are required to perform complex tasks under high stress and uncertainty. One of the major challenges of aviation medicine is the selection of suitable candidates to serve as pilots. The vagal nerve is a crucial moderator of stress responses, and its activity (indexed by heart rate variability, HRV) reflects psycho-physiological resilience and has been shown to predict performance in various settings. However, its predictive value in pilot training has not been examined. This study examined the relationship between HRV and success in an intensive and long pilot course.

**Methods:**

In a historical prospective study, we derived an HRV parameter (RMSSD) from a 10-second ECG of 169 male and 16 female candidates attending a 3-year pilots’ course. The ECGs were performed 2–3 months before the courses. The predictive validity of other routinely obtained measures was also considered. Data were analyzed in two ways. First, we analyzed the entire sample using t-tests. Then, significant predictors of success and HRV were entered in a multivariate logistic regression. Second, we focused on a smaller sample of paired candidates (passed vs. failed), matched on significant predictors, and then examined differences in HRV between these groups using a paired t-test.

**Results:**

High RMSSD significantly predicted the completion of the pilot course in a multivariate logistic regression. RMSSD and the selection test formula score were the only significant predictors. In the paired matched sample, candidates who passed the course had significantly higher initial HRV (M = 121.30ms) compared to those who failed (M = 84.31ms; t(25)= 1.78, p < 0.05).

**Conclusion:**

The current study supports the predictive value of HRV for aviation selection. Given the high cost of training operational pilots and the physical and mental burdens they undergo, improved accuracy of the selection processes may be crucial.

Operational pilots are required to perform complex tasks under high stress and uncertainty [1]. This requires coordination between the brain and several physiological systems. This study will review interdisciplinary evidence showing that the vagal nerve reflects such integration and predicts completing a highly competitive pilot training course. During in-flight training, pilots are required to execute cognitive processes involving visual and auditory information, decision-making based on received and previous knowledge and working memory [2]. Agge and others [3] asked 1092 U.S. Air Force staff to rank the cognitive abilities necessary for pilots’ work. The top 3 abilities were situational awareness, spatial orientation, and task management/multi-tasking. Numerous studies have demonstrated a positive correlation between HRV and cognitive abilities required in flight missions (e.g., [4–7]).

The vagus (the 10th cranial nerve) is the main nerve of the parasympathetic branch of the autonomic nervous system (ANS). Its activity moderates the intensity and duration of the stress response (e.g., blood pressure, cortisol, and inflammation) [8]. Heart rate variability (HRV) is a measure that represents the changes in the interval between heartbeats and can be used to assess and monitor vagal nerve activity [9–11]. HRV can be measured in three conditions: at rest, during, and after activity. The tonic index (Tonic HRV) represents a measurement during rest or baseline measurement [11]. The changes in the intra-beat interval at rest mainly reflect the vagal activity, and higher HRV reflects greater vagal activity [12].

When interpreting HRV data, the observed physiological response must be considered in relation to the time point and context of the measurement. For example, during rest, high HRV is considered adaptive in most cases [11], and many researchers refer to HRV at rest as a measure of adaptivity to different challenges [13]. Resting HRV is positively correlated with cognitive flexibility and inhibition of behaviors [6]. Furthermore, HRV positively correlates with brain activity in areas essential for attention-switching [14,15], decision-making, and emotional regulation [16]. Alongside these psychological and cognitive abilities, high resting HRV is also positively correlated with better fitness and physical performance [17,18]. These abilities are crucial for adequate performance in operational settings.

In the general military context, the predictive value of HRV in relation to performance in extreme conditions is contradictory. In three prospective studies, low HRV just before intensive military training predicted better performance [19]. In contrast, in another study, high HRV taken two months before intensive military training predicted better performance [20]. The timing of the measurement can explain the inconsistency in these results, which will be discussed later. The correlation between HRV, performance in extreme conditions, and cognitive abilities (e.g., [7]) suggests that HRV can serve to identify suitable candidates for operational positions such as pilots, which involve high cognitive and motoric capacities. In the aviation setting, a recent review found an increase in sympathetic activity during flights, the sensitivity of HRV to flight demands, and ANS changes during recovery [21].

Exposure to stress can impair cognitive capabilities (e.g., [22–25]). In contrast, cognitive resilience is the “capacity to overcome the negative effects of setbacks and associated stress on cognitive function or performance” [26] (stall 2008, p. 260). One of the significant challenges that preoccupied the military and the research literature is the selection of suitable (“resilient”) candidates to serve as pilots. The neurovisceral theory assumes that the positive relationship between vagal activity and performance will be preserved in stressful situations such that people with high HRV at rest will perform well even in challenging conditions, thus reflecting resilience and adaptivity [7,27].

One of the key components for military success in a modern battlefield is air superiority and a high-performing air force with high-quality pilots [28]. Military pilot training is expensive (approx. 5–10 million dollars per pilot [29]) and long (e.g., 3 years). The high costs of the training and the aircraft, the complexity of training, and the need to maintain the safety of the candidates lead armies to optimize the selection procedure in pilots’ courses [29–31].

Currently, the Israeli Air Force uses a multi-step selection process that includes four stages of selection (See Fig 1). These stages include a physical fitness test, a psychometric test, a psychological interview, and several flying simulator tests. Candidates who pass these stages are invited to a 5-day selection test examining their physical and cognitive abilities under relevant stress. At the end of these 5 days, a formula is used to calculate candidate performance and predict their chances of passing the course.

**Fig 1:**
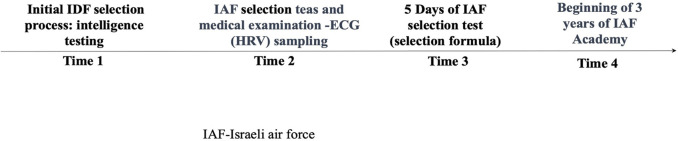
IAF selection flow chart.

The relation between HRV at rest and pilots’ performance was hardly examined despite its relevance. In one study, Cao and others [32] demonstrated a positive relationship between HRV and pilots’ performance in a simulator. Recently, a team from the Aero Medical Institute in Belgrade found that higher resting HRV predicted better G+ tolerance and proposed that HRV may be an additional tool in pilot selection procedures [33]. It should be noted that several aspects of the existing selection process depended on the subjective evolution of external evaluators. Therefore, there is a need for an objective and rapid measure that reflects both physical and mental capabilities. As mentioned above, HRV meets these criteria. The studies reviewed above suggest that HRV may predict the completion of pilots’ courses since it is theoretically and empirically related to executive functioning and stronger psychobiological resilience, all pivotal for pilots’ adequate performance.

To the best of our knowledge, HRV has never been tested as a predictor of success in a military pilot course. The purpose of the present study was to examine the ability of HRV to predict success in the Israeli Air Force pilots’ course, an enduring and highly demanding course. We hypothesized that participants with initially higher levels of resting HRV before the course would pass at a greater rate than those with low levels of resting HRV. These differences were expected to endure after controlling for the effects of other background factors and predictors. On the practical level, the study’s results could have implications for identifying the most suitable candidates for pilot courses.

## Materials and methods

### Participants and procedure

The participants were candidates for one Israeli Air Force pilot training. We analyzed the records of 185 male and female candidates. Like other militaries, the Israel Defense Forces (IDF) do not share data regarding success rates. We arrived at this final number after excluding cases with unreadable ECG (due to military confidentiality issues, we cannot provide the initial number of cases).

### Study design

The study was historical prospective, all data were accessed anonymously between 10/01/2024 and 12/04/2024, and the authors have no access to candidates’ personal information. HRV was derived from ECG in files of medical tests performed 2–3 months before the courses (T1), which were used to predict the outcome of the candidate’s performance at the pilot’s course over three years (T2). This time window (2 months before training) was found to predict better performance in military settings [20,34]. All ECGs were performed using the same device in the Israeli Air Force Aeromedical Center facilities (**Nihon Kohden 9020**), and only a 10-sec ECGS was available.

The ECG strips from the selected sample were manually scanned and analyzed using a method used in our laboratory and reported in other studies [35]. The analysis of the ECG charts was done by the first author (YK), who was blind to the performance of the candidates. The study was approved by the IRB of the IDF Medical Corps protocol number 2352−2023.

### Physiological measures

The HRV measure was taken from 10-second ECG charts. **We focused on the time-domain** measure of the root mean square of successful difference (RMSSD). Similar to other HRV and selection studies, all ECGs were taken 2–3 months prior to the selection [20,34].

#### RMSSD.

This measure represents the root mean square of the differences between successive heartbeats. The index assesses vagal tone and is less affected by breathing [11,36]. RMSSD is a reliable index for estimating HRV from ultra-short ECG charts, and RMSSD from 10-second recordings is highly correlated with 5-minute measurements (e.g., [37]).To determine RMSSD, the ECG charts from the selected sample were manually scanned and analyzed using a method adopted in our laboratory. It was reported in other studies [34,35]. First, we scanned paper ECGs and saved them as PDF files, verifying and maintaining the original time scale. Then to measure RMSSD, we used the Adobe Acrobat software ruler (www.adobe.com) to manually measure inter-beat intervals on an HRV basis. We obtained high inter-rater reliability levels (n = 8, r = 0.9) in these measures. RMSSD values are presented in milli-seconds (ms) and heart rate in beats per minute (BPM).

### Cognitive measures

#### Intelligence testing.

The intelligence test has been found to predict performance in the military setting [34]. This test examined the candidate’s cognitive ability and consisted of a series of cognitive tests, including verbal, quantitative, and spatial components. The score ranges from 10 to 90 in 10-point intervals [38,39]. IDF has used intelligence testing since the 1950s, and the IDF Behavioral Science Center developed the current version. This test is mandatory for all IDF candidates in the first stage of their selection process.

### Other measures

#### The selection test formula.

This score weighs the candidate’s performance in the five days of the selection test and specifies who will start the course [40]. For reasons of confidentiality, the calculation formula cannot be detailed.

### Passing the course

The pilot course includes five phases, but we focus only on the final outcome—whether the entire course passes or fails. The pilot course in Israel includes basic military training (including an officer course), basic pilot training, and advanced training depending on the aircraft to which he will be assigned. In addition, during the course, all trainees study for a bachelor’s degree. Throughout the course, the trainees’ flying skills are tested, and a trainee who does not meet the requirements is dismissed. The course is completed by trainees who have met all the training requirements. The statistical analysis was based on the number of initial candidates for the outcome.

#### Statistical analyses.

We used a two-stage approach to analyze this study’s data. First, we analyzed the entire sample using t-tests to examine differences in continuous measures between candidates who passed and those who did not. Then, significant predictors of success and HRV were entered in a multivariate logistic regression using the enter approach. Second, we focused on a smaller sample of paired candidates (passed the entire course versus failed), matched on significant predictors, and then examined differences in HRV between these groups with a paired t-test. The second analysis enabled us to methodology control for other predictors and examine with more statistical power the predictive role of HRV in pilot course competition independent of the matched variables. The statistical analyses were conducted using IBM SPSS Statistics 27.0 software. The significance level was set at p < 0.05 one-tailed.

## Results

The sample included 169 males and 16 females aged between 18 and 23 (mean 18.92, s.d-0.76), and 30 candidates completed the entire course. Participants’ mean BMI was 22.04 (s.d 2.24), and the mean weight was 67.92 kg (s.d 8.55).

A t-test for independent samples was performed to test the first hypothesis. The mean initial HRV in those who later passed the pilots course (M = 115.15ms, s.d = 95.8ms) was significantly higher than in those who failed (M = 82.74ms, s.d = 49.72ms), t(183)= 2.731, p < 0.001). As in many other studies, the RMSSD data were not normally distributed. Hence, a log transformation was applied as recommended (Laborde et al., 2017). After the transformation, RMSSD was still significantly higher in those who passed the course t (183) = 1.88, p < 0.05. For comprehensibility, the means are presented without the transformation. Additional T-tests were performed for the other psychological and physiological predictors concerning the course completion (see Table 1).

**Table 1:** Means, standard deviations (SD), and t-tests for predictor variables in relation to course completion.

	Passed the course		Did not pass the course		*t (pdf)*	*sig*	*Cohen’s d*
	*M*	*SD*	*M*	*SD*			
Intelligence score	84.00	8.55	80.38	9.45	1.94 (183)	*p* < 0.05	*0.38*
Selection test formula	29.71	2.90	28.17	1.76	−1.938(183)	*P* < 0.05	*0.77*
RMSSD	115.15 ms	95.8 ms	82.74 ms	49.72 ms	1.88(183)	*p* < 0.05	0.54

RMSSD: Root Mean Square of Successive Differences between normal heartbeats

M: mean MS: Milliseconds; SD: Standard deviation; df: degree of freedom; sig: significant.

Multivariate logistic regression was used to test the predictive value of HRV for success in the course alongside the intelligence testing score and selection formula. RMSSD (O.R = 1.008, 95% CI: 1–1.001, p = 0.011) and selection test formula scores (O.R = 1.36, 95% CI: 1.12–1.66, p = 0.002) were the only significant and independent predictors of course completion (see Table 2).

**Table 2:** Multivariate logistic regression of predictors of course completion.

Variable	B	S.E.	Sig.	O.R	Lower	Upper
Intelligence Score	.031	0.27	0.248	1.03	.979	1.08
Selection test formula	.312	0.1	0.002	1.36	1.12	1.66
RMSSD	.008	0.003	0.011	1.008	1	1.01

RMSSD: Root Mean Square of Successive Differences between normal heartbeats; B = Beta coefficient; S.E: Standard error; Sig: Significance; O.R-odds ratio; n = 185.

### Matched sample

**Fig 2:**
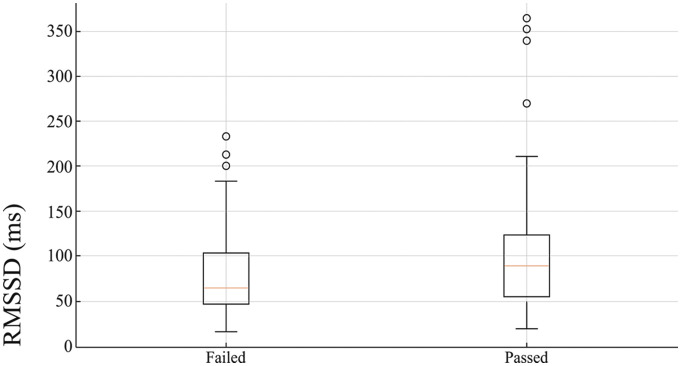
The mean heart rate variability (HRV) of cadets who later passed or failed the pilot course after matching for significant confounders. RMSSD: Root Mean Square of Successive Differences between normal heartbeats**; MS- Mili Seconds.**

In order to increase statistical power beyond the effects of significant cofounders, we decided to perform a matched-sample analysis. Candidates were paired and matched on significant cofounders: Intelligence testing score and selection formula. In this matched analysis, the mean initial HRV of those who passed the pilot course (M = 121.30ms, s.d = 61.48ms) was significantly higher than that of those who failed (M = 84.31ms, s.d = 12.05ms) t(25)= −1.78, p < 0.05 (see Fig 2).

## Discussion

In the present study, the main finding is that initial HRV levels were significantly higher in candidates who later passed the course. This is in line with our hypothesis and with the findings of other studies in the military selection field [20,34]. HRV was still a significant predictor after controlling for other predictors and in the matched sample. The pilot’s academy requires candidates to maintain physical, psychological, and cognitive functions in very stressful settings. Physiological indices appear to have a high predictive value in the selection phases requiring high-intensity fitness. This value decreases during the training phases that require learning new skills [41]. High resting HRV reflects cognitive, psychological, and physiological abilities [6,8,17].

The present study aimed to examine the ability of the vagal nerve activity indexed by HRV to predict the completion of an Air Force flying course. The selection of pilots in aviation is a significant challenge due to training costs and high risks. In the present study, HRV was a significant predictor of completing the pilot course after statistically controlling for confounders in the entire sample. To increase the statistical power, we used a matched pair analysis (we matched pass and fail candidates on significant predictors). HRV was still a significant predictor of course completion in this stricter analysis.

To the best of our knowledge, this is the first study to examine the relationship between initial HRV and completing a three-year military pilot course. In previous studies in the military, low HRV predicted better performance [19], while in another study, high HRV before intensive military training predicted better performance [12]. We assumed that the timing of HRV measurement could explain the differences between studies. In our study, as in Stanffil et al. [20], HRV was taken approximately two months before a highly stressful training. The measuring of HRV just before an extreme stressor may reflect a sympathetically driven (state) reactive HRV. In contrast, HRV measured long before a stressor may reflect parasympathetically driven resting (trait) HRV [42].

Military pilot training includes extreme physiological and cognitive challenges. The first stages of the pilot course include mainly physical stressors (e.g., survival skills), while the more advanced stages require cognitive abilities (e.g., multi-tasking, situational awareness). As shown above, HRV may combine both abilities, which could explain our results. Our results are in line with neurovisceral theory. The neurovisceral theory [7,43] which assumes that the positive correlation between HRV and these abilities remains stable under stress. Thus, people with high resting HRV will perform better under stressful conditions, such as pilot’s academy.

The ability to rest HRV to predict performance in the long run (3 years course) could be explained in several ways. People with high resting HRV recover physiologically faster from stress than people with low HRV [8]. HRV positively correlated with cognitive abilities (cognitive flexibility and inhibition) [6]. In addition, HRV positively correlates with brain activity in frontal regions, which are essential for attention switching [14,15]. Furthermore, HRV correlates with better physical fitness [17]. Finally, at the psychometric level, resting HRV is considered a relatively stable measure, e.g., ICC = 0.89 over 24 months [44].

We assume that all these capacities related to HRV could explain the relationship between HRV and course completion.It should be noted that in the current study, the intelligence score and the selection formula were also significant predictors of course completion. Recent data have demonstrated that the combination of psychological and physiological predictors improves the predictive value of success in intensive military training [45]. On a practical level, our results suggest that HRV may improve the identification of the most suitable candidates for pilot courses.

The current study has several limitations. First, it was a historical prospective correlational study. Thus, we had limited control over the measurement conditions of HRV, and we cannot infer any causal relations. In addition, specific measures that could impact HRV (such as perceived stress, exercise, and coffee) were not taken. Third, the HRV measurements were taken from an ultra-short ECG (10 sec). Therefore, only HRV time domain indices were taken. Similar to the other studies mentioned above, the HRV measurement was taken only at one point. Along with the need to optimize the screening processes in pilot courses and the potential of HRV, follow-up studies are needed to understand the predictive value of HRV in the various stages of training and selection. In future studies, a prospective design is needed to measure HRV at several time points along the course, with the assessment of perceived stress. Future studies should also examine whether cognitive functions explain the relationship between HRV and pilot course completion.

In conclusion, despite these limitations, our study supports the predictive value of HRV in pilot training, possibly because it reflects adaptive psychological and physiological dimensions. These results may be used in the selection process for operational positions in military and civilian settings, which could reduce costs and prevent adverse health outcomes. It should be noted that in the current study, we propose using HRV in the screening phase after psychological screening and other physiological indicators to identify the candidates who have the physiological resilience to cope with stressors. Given the high cost of pilot training and the burden they undergo, more precise selection procedures are needed to prevent errors in recruitment. Therefore, measuring HRV may be an objective and evidence-based way to identify soldiers who may have the physiological resilience to cope with stressors since it predicts passing or failing in highly demanding training. The timing of the HRV measurement is of critical importance. If HRV were to be used for selection purposes, it must be measured in a state of low or no stress, out of the context of the selection procedures.
